# The effectiveness of using situational awareness and case-based seminars in a comprehensive nursing skill practice course for undergraduate nursing students: a quasi-experimental study

**DOI:** 10.1186/s12909-024-05104-y

**Published:** 2024-02-06

**Authors:** Yuanhao Sun, Xiangdong Li, Haiyang Liu, Yuqing Li, Jiaofeng Gui, Xiaoyun Zhang, Xiaoping Li, Lu Sun, Lin Zhang, Congzhi Wang, Jing Li, Mingming Liu, Dongmei Zhang, Jingyi Gao, Xuefeng Kang, Yunxiao Lei, Ting Yuan

**Affiliations:** 1 Department of Graduate School, Wannan Medical College, Wuhu, An Hui China; 2 Department of Gerontology, Yijishan Hospital, the First Affiliated Hospital of Wannan Medical College, Zheshan West Road, Yijishan District, Wuhu City, Anhui Province People’s Republic of China; 3 Student Health Center, Wannan Medical College, 22 Wenchang West Road, Higher Education Park, Wuhu City, An Hui Province People’s Republic of China; 4 Department of Emergency and Critical Care Nursing, School of Nursing, Wannan Medical College, 22 Wenchang West Road, Higher Education Park, Wuhu City, An Hui Province People’s Republic of China; 5 Department of Internal Medicine Nursing, School of Nursing, Wannan Medical College, 22 Wenchang West Road, Higher Education Park, Wuhu City, An Hui Province People’s Republic of China; 6 Department of Surgery Nursing, School of Nursing, Wannan Medical College, 22 Wenchang West Road, Higher Education Park, Wuhu City, An Hui Province People’s Republic of China; 7 Department of Pediatric Nursing, School of Nursing, Wannan Medical College, 22 Wenchang West Road, Higher Education Park, Wuhu City, An Hui Province People’s Republic of China; 8 Department of Nursing School, Wannan Medical College, Wuhu, An Hui China; 9 Department of Gynecology and Obstetrics Nursing, School of Nursing, Wannan Medical College, 22 Wenchang West Road, Higher Education Park, Wuhu City, An Hui Province People’s Republic of China

**Keywords:** Situational awareness, Case-based seminars, Self-directed learning, Professional identity, Academic self-efficacy

## Abstract

**Background:**

Nurses play an important role in healthcare development. The increasing demands for nurses mean that nursing schools at the undergraduate level have the responsibility to ensure patient safety and quality care through a well-designed curriculum. This research aimed to evaluate the effect of the teaching method combined with situational awareness and case-based seminars in a comprehensive nursing skills practice course on the level of self-directed learning, professional identity, academic self-efficacy, theoretical scores, practical scores, teaching satisfaction, and student competence among nursing students.

**Methods:**

The research population comprised was of the grades of 2019 and 2020 at Wannan Medical College in Anhui Province, China (*n* = 169, response rate 77.88%). The observation group from grade 2020 used the teaching method combined with situational awareness and case-based seminars, whereas the control group from grade 2019 used the traditional teaching mode. General information, self-directed learning, a professional identity, and academic self-efficacy were compared between the two groups. This research used means and standard deviations, chi-square, the Shapiro–Wilk test, and an independent sample t-test for statistical analyses.

**Results:**

Compared with the control group, the total scores for self-directed learning, professional identity, and academic self-efficacy were higher in the observation group (78.80 ± 7.89 vs 60.21 ± 7.44, 63.39 ± 7.87 vs 52.35 ± 7.68, and 22.31 ± 3.30 vs 21.28 ± 2.31, respectively, with *P* < 0.05 for all scores). More significant improvements were made in the observation group on the level of theoretical scores (81.39 ± 3.32 vs 76.28 ± 5.90) and practical scores (93.32 ± 4.70 vs 90.67 ± 5.09) (*P* < 0.05). Meanwhile, teaching satisfaction, which includes teaching method (66/18 vs 32/53) and teacher-student interaction (72/12 vs 34/51), and student competence, which includes team cooperation (67/17 vs 39/46), critical thinking (60/24 vs 31/54), and communication skills (67/17 vs 38/47) after the intervention (*P* < 0.05). There was no significant difference in social persuasion (*P* > 0.05).

**Conclusion:**

The teaching method combined with situational awareness and case-based seminars in a comprehensive nursing skills practice course has the potential to improve the level of self-directed learning, professional identity, and academic self-efficacy, and it increases theoretical scores, practical scores, teaching satisfaction, and student competence.

## Background

Nurses play an important role in healthcare development. Nursing constitutes the largest proportion of hospital staff in most countries, so ensuring an adequate number of skilled nurses is vital for quality nursing services [1]. A good transition from nursing students to nurses may encourage professional nurses to face numerous challenges and affect the long-term retention of nurses [2]. Therefore, nursing students need to master the relevant theoretical and practical skills of the nursing discipline. Then they will be capable of handling nursing emergencies and solving nursing problems effectively [3]. When it comes to the theory and practice of clinical nursing, engaging students in the complexities of medical care is challenging. The increasing demands for nurses mean that nursing schools at the undergraduate level have the responsibility to ensure patient safety and quality care through a well-designed curriculum [4].

Situational awareness can be defined as the perception and understanding of elements of the environment in a given volume of space and time, as well as the projection of their future status [5]. Endsley defined situational awareness as “a person’s mental model of the world around them” in connection with aviation [6]. Situational awareness is a part of information processing that follows perception and influences decision-making as well as action execution [7]. In the simplest terms, situational awareness allows an individual to see the big picture of a situation while dealing with the details and issues as they arise [8]. Situational awareness has been recognized as a crucial component of medical practice in the field of healthcare, encompassing several cognitive abilities such as perception, comprehension, reasoning, and meta-cognition [9]. Situational awareness has four hierarchal levels, perception, comprehension, projection, and following the path of the best outcome for the patient [10]. With the changing learning environment, it is gradually attracting more and more interest. When situational awareness is lacking, judgment is unavoidably compromised, maybe with negative consequences [11]. Multiple studies have shown that a lack of situational awareness, including poor team communication and insufficient knowledge, can lead to safety issues and adverse events [10, 12–15]. Situation awareness is a necessary patient safety skill. Nurses are in the best position to identify early clinical deterioration since they spend more time at the patient's bedside than members of other healthcare disciplines [16]. Inadequate situation awareness is demonstrated by failure to detect patient deterioration in the early stages. The World Health Organization identified insufficient situational awareness as the principal factor linked to subpar clinical outcomes and suggested incorporating this knowledge into undergraduate medical education [17]. At the University of Dundee, non-technical skills such as situational awareness have been considered as training to be included in the undergraduate medical students [18]. In the operating theatre and intensive care setting, situational awareness was also widely used for better treatment results [13, 19]. Thus, it could be necessary and beneficial to include situational awareness in a comprehensive nursing skills practice course.

Using case-based learning is crucial for teaching medical students [20]. In case-based courses, medical knowledge is applied to clinical cases, allowing students to apply clinical reasoning and decision-making during various phases of the clinical case management process, including clinical examination, diagnosis, and treatment [21]. The present research focused on case-based seminars, a student-centered education based on the case. Spruijt’s research indicated that seminars help students become active participants in their learning by enabling them to take part in discussions under the direction of a lecturer [22]. This process allows the lecturer to observe and guide students through the various stages of the comprehensive nursing skills practice course by asking questions, explaining, and encouraging discussion among students. The purpose of case-based seminars is to encourage students to actively participate in class, prepare them for their role as nurses, and bridge the gap between foundational medical knowledge and clinical work. By using this method, students and teachers can maximize their academic potential, improve their understanding of research problems, and match their “teaching” with their “learning” to achieve the best teaching results [23].

Self-directed learning means the process of taking initiative in evaluating learning needs, setting learning goals, selecting and implementing appropriate learning strategies, and evaluating learning outcomes either with or without the assistance of others [24]. Self-directed learning is an effective method for nursing students to apply their skills in a variety of clinical settings after graduation, adapt to the rapidly changing healthcare environment, and demonstrate their professional skills [25]. It has been discovered that self-directed learning works well for nursing education. For instance, self-directed learning was found to be significantly associated with academic achievement in a study of Turkish nursing students [26]. The term “professional identity” describes one's professional self or self-concept in their work [27]. Nurses’ professional identity occurs throughout almost their entire careers, which makes education at the undergraduate level especially important. Nursing students receive a great deal of theoretical knowledge and professional skills during their educational years. These experiences help them to increase their professional self-confidence, improve their sense of professional self-efficacy, promote the desire for continuous learning, and encourage the internalization of nurse’s values [28]. A key element in the development of nursing practice and education is professional identity. It was discovered that a willingness to become a nurse was strongly correlated with having a positive professional identity [29, 30]. The concept of academic self-efficacy refers to students' belief in their ability to achieve their educational goals [31]. Based on Bandura’s self-efficacy theory, human achievement is determined by the interaction between behavior, beliefs, and environment [32]. With high self-efficacy, students are more likely to accept challenging tasks, demonstrate greater motivation, and persist in the face of difficulties. In contrast, students with low self-efficacy tend to be underconfident in their educational abilities and struggle to complete their assignments [32, 33]. It’s significant to note that self-efficacy affects not just performance and success but also help-seeking and motivational behaviors that promote learning [34]. It is obvious that the nursing field as a whole and nursing students in particular benefit from the development of these abilities.

To improve self-directed learning, academic self-efficacy, and professional identity in undergraduate nursing students, a comprehensive nursing skills practice course has been designed that integrates situational awareness and case-based seminars to improve competence. Previous research focused more on the effects of problem-based learning, case-based learning, or task-based learning on the competence of nursing students [35–40]. To date, a limited amount of research has explored the effects of situational awareness and case-based seminars in a comprehensive nursing skills practice course. It is essential to choose a pedagogical strategy for nursing students to guarantee that all nursing graduates possess the skills required for both theory and practice.

Therefore, this study aimed to evaluate the effect of the teaching method combined with situational awareness and case-based seminars in a comprehensive nursing skills practice course on the level of self-directed learning, professional identity, academic self-efficacy, teaching satisfaction, and student competence among nursing students.

## Materials and methods

### Study participants and procedure

**Fig. 1:**
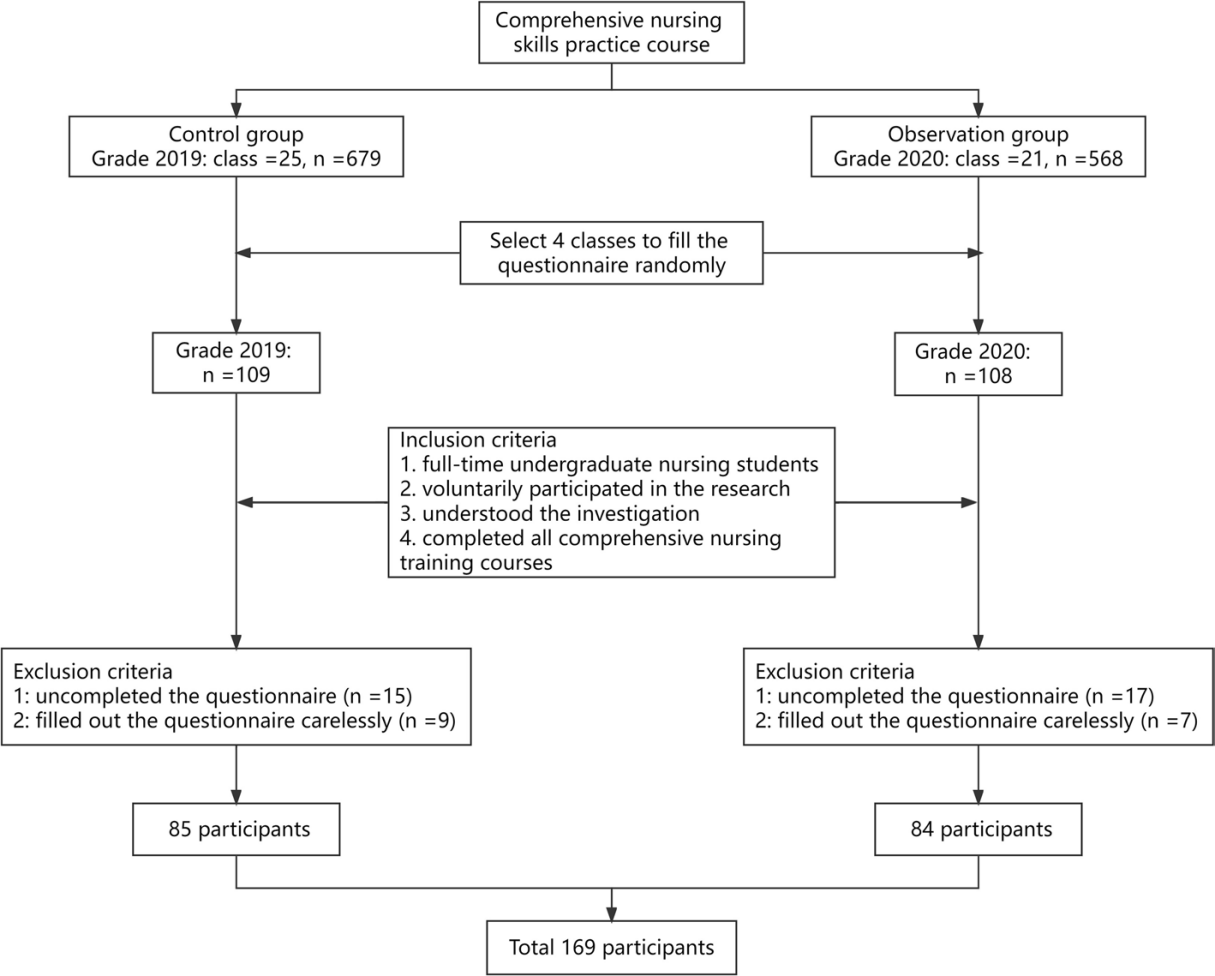
Study procedure

This is a simple random sample research and this research used the parallel design method for nursing students from the grades of 2019 (*n* = 679) and 2020 (*n* = 568) from a medical college located in Anhui Province in China. The control group was taught from September 2021 to January 2022, while the observation group was taught from September 2022 to January 2023. Then the random number table method and simple random sampling to select four classes of students, each comprised of approximately 27 students, to fill out the questionnaire. Questionnaires will be distributed after each practice course, completed voluntarily, and returned one week later. Among them, the control group had 109 questionnaires, and the observation group had 108 questionnaires. The inclusion criteria for the participants were: (1) full-time undergraduate nursing students; (2) voluntarily participated in the research; (3) understood the investigation; and (4) completed all comprehensive nursing training courses. The exclusion criteria were: (1) uncompleted the questionnaire; and (2) filled out the questionnaire carelessly. The purpose of the survey will be explained to all students, and they could withdraw from the research at any time. It would not affect their academic scores in future studies. Students will fill out the questionnaire within a week after the course, which can effectively reduce the occurrence of information bias. Feedback questionnaires were gathered to compare student learning under different teaching approaches they experienced. Finally, after those who met the exclusion criteria were removed, 77.88% (169 out of 217) of the staff were included in the statistical analyses (Fig. 1). The grade of 2019 had their theoretical and practical exams in January 2022. For the grade of 2020, the exams are conducted in January 2023.

### Ethical consideration

Approval to conduct the research was obtained from the Ethics Committee of the School of Nursing at Wannan Medical College (no.20210008.10) and was performed as prescribed by the Declaration of Helsinki.

### Teaching methods

Both the observation group and control group were instructed by the same experienced lecturers with the same syllabus and cases. All the students attended a comprehensive nursing skills practice course for 160 min.

### The control group

**Fig. 2:**
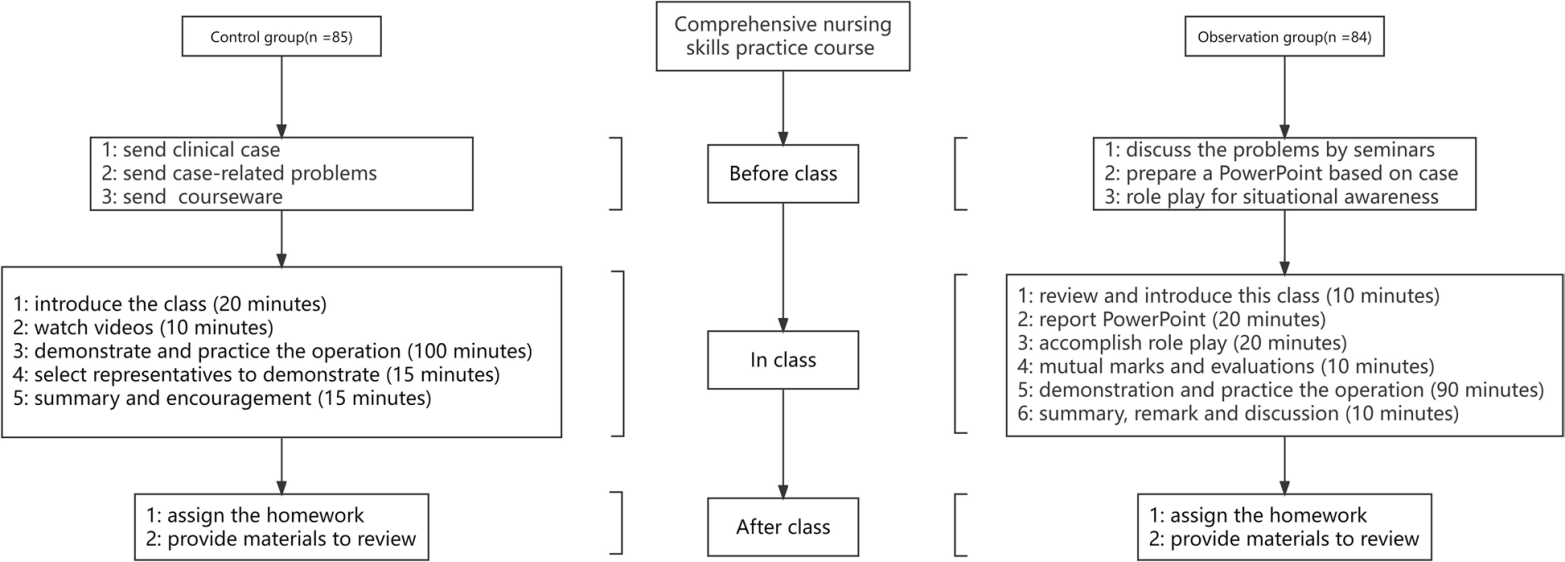
Overview of the study design

This method is regarded as a teacher-centered educational approach, with the knowledge being proactively transmitted by the lecturer and passively received by the students. The relevant clinical case, case-related problems, and courseware would be sent by the lecturer ahead of schedule to the students. These can reserve valuable in-class time because it is not easy for students to achieve the teaching objectives only with textbooks. In the class, the lecturer would provide a detailed introduction to the class within the theoretical framework based on the syllabus via PowerPoint or face-to-face communication (20 min). Secondly, with the visual information easier to absorb and engage, each student needed to watch videos that were related to the teaching content to promote their understanding of the curriculum (10 min). Thirdly, the lecturer will personally demonstrate the experimental procedure and remind the participants of the details that were easily ignored, followed by all the participants being divided into small groups (5–6 students per group) to practice the operation (100 min). Soon afterward, a representative from each group was selected to demonstrate the operation, which can detect the effect of the exercise (15 min). Finally, the lecturer summarized the class, going over the questions raised during the discussion, reviewing the main points from the lecture, and encouraging students to remember and learn (15 min). After the class, the lecturer would give the review materials and assign homework, and then the students were required to finish and hand in it within one week (Fig. 2).

### The observation group

This method, which is combined with situational awareness and case-based seminars, is regarded as a student-centered approach that allows full play for students’ subjective initiative. Before the class, the lecturer would provide significant materials for the students’ preview, which included clinical-based case and case-related problems. All the students were divided into four groups, and each group was comprised of approximately six to seven students. Meanwhile, each group was required to complete three assignments: (1) analyzing and discussing the questions raised by the lecturer and using the knowledge to solve them by way of seminars; (2) preparing a PowerPoint presentation for the report in the class that can cover the keys to the case and the course topics; and (3) planning situated nursing activities that require students to play roles such as nurses, patients, or family members for better situational awareness, practice nursing skills, and patient-centered communication. In their free time, students were needed to discuss and make a division of labor, including searching for relevant information, designing the role play, practicing the correlated experimental operation, and consulting the literature for the particularly challenging problems. In the class, step 1, the lecturer would briefly review the last lecture, introduce the main content of this time course, and indicate the key points of knowledge in students’ learning (10 min). Step 2, a five-minute or so PowerPoint presentation will be reported by a student representative from each group to analyze the case and answer the question (20 min). Through this process, the lecturer could evaluate the mastery degree of the learning content. Step 3, each group was required to complete their role play to develop situational awareness based on the case, which included common clinical scenes (20 min). By the way, their team cooperation and clinical communication competence will also be enhanced. Step 4, the different groups will rate each other on how well the PPT was reported, how well they answered the questions correctly, and how well the actions during the role-play complied with the nursing scoring criteria and moral and ethical requirements (10 min). Also, as an assessment of situational awareness, the teacher will score the students’ ability to predict the next steps in the patient’s disease and to formulate appropriate nursing care. Students could ask questions through interactive dialogue. This learning pattern allowed them to become participatory and joyful. The class was not boring but rather full of enthusiastic engagement and expectation. For step 5, relevant operational demonstration, which was needed in the case, will be given by the lecturer to bridge the gap in knowledge between the classroom and the actual clinical practice. Next, students will practice the operation (90 min). The lecturer will be able to help them if they have problems during practice. For step 6, the lecturer would make a summary, remark, and discuss the positive aspects of this class, as well as the methods for improvement that were undertaken (10 min). Meanwhile, students were encouraged to timely review to consolidate the knowledge that can assist them in working out problems flexibly and correctly. After the class, the lecturer provided a recorded video, several relevant questions, and supplementary study materials to encourage students to review in time. Students can go over what they have learned from these, and the answers will be issued after a few days. At the same time, all of the students needed to complete a lab report based on this class (Fig. 2).

### Assessment instruments

#### Demographic questionnaire

The social-demographic research collected information on gender, living area, student leader, only-child, average age, and last semester's final scores.

#### Self-directed learning for nursing students

The self-rating scale was developed by Su-Fen Cheng in 2001 and was used to evaluate autonomous learning ability [41]. The scale contains 20 items, which are divided into 4 dimensions, including learning motivation for 6 items, planning and implementing for 6 items, self-monitoring for 4 items, and interpersonal communication for 4 items. Each item used a five-point Likert scale coded from 1 “strongly disagree” to 5 “strongly agree.” All the items are positively stated. The total points on the scale ranges from 20 to 100, and a higher score indicates a higher level of learning ability. The scale has good content validity and structure validity. The Cronbach’s alpha of the above four dimensions is 0.801, 0.861, 0.785, and 0.765, respectively. In this research, the Cronbach’s alpha is 0.951.

#### Professional identity questionnaire for nursing students

The self-rating scale was designed by Hao Yufang’s team [42]. The scale is composed of five dimensions, with 17 items in total. Six items for checking for professional self-concept (1, 6, 9, 11, 16, 17), four items for benefit of retention and risk of turnover (5, 8, 10, 14), three items for social comparison and self-reflection (7, 13, 15), two items for independence of career choice (4, 12), and two items for social persuasion (2, 3). A five-point Likert scale (1 = strongly disagree; 2 = disagree; 3 = neutral; 4 = agree; 5 = strongly agree) is used, and item 12 is negatively stated. A higher total score means a higher level of professional identity. The Cronbach’s alpha value in this research was 0.916.

#### Academic self-efficacy scale

The academic self-efficacy scale, compiled by Hellriegel and translated into a Chinese version by Wenzhao Yu in 2001, aims to measure the students’ self-efficacy in terms of academic achievement. Using the five-rating method, the scale comprises 7 items. 1, 3, 6, and 7 items are positively stated, using a five-point scale coded from 1 “strongly agree” to 5 “strongly disagree,” and “S1” was used to represent the sum of the scores. For that, 2, 4, and 5 items are negatively stated, and responses range from “strongly agree” to “strongly disagree”. Scores range from 5 to 1 and “S2” was used to represent the sum of the scores. Finally, the score of academic self-efficacy was expressed by the sum of S1 and S2. Higher scores designated better academic self-efficacy. The Cronbach’s alpha value in this research was 0.755.

#### Statistical analysis

The study used IBM SPSS version 25.0 (Chicago, IL, USA) for all statistical analyses. Means and standard deviations are used to present the distribution of continuous variables (average age, last semester's final scores). The distribution of categorical variables (gender, living area, student leader, only-child) was analyzed by chi-square. Shapiro–Wilk test was used to verify the normal distribution of the variable. The independent sample t-test was used to analyze the discrepancy between two sets of variables. *P* < 0.05 was considered statistically significant for all statistical analyses.

## Result

### General baseline characteristics for nursing students

As shown in this research, it is based on the basic nursing student characteristics in the observation group and the control group. There was no significant difference between the two groups in terms of gender, living areas, student leaders, only-child, or average age (*P* > 0.05). Additionally, the last semester’s final scores in the observation group (78.13 ± 6.82) and control group (78.28 ± 5.18) were considered to be not statistically significant (*P* > 0.05) (Table 1).

**Table 1:** General baseline characteristics(*N* = 169)

Variables	Observation group	Control group	t/χ^2^	P
	(*n* = 84)	(*n* = 85)		
Male/Female	27/57	31/54	0.35	0.554
Living areas(Countryside/City)	51/33	48/37	0.31	0.575
Student leaders(Yes/No)	30/54	22/63	1.92	0.166
Only-child(Yes/No)	26/58	29/56	0.19	0.661
Average age($\overline{{\varvec{\chi}} }$±s)	21.38 ± 0.89	21.38 ± 1.00	0.03	0.975
Last semester's final scores($\overline{{\varvec{\upchi}} }$±s)	78.13 ± 6.82	78.28 ± 5.18	0.16	0.871

### Self-directed learning for nursing student

Compared with the control group, the students that were in the observation group obtained higher scores in learning motivation (23.58 ± 3.47 vs 18.29 ± 2.93), planning and implementing (23.69 ± 2.69 vs 17.88 ± 2.46), self-monitoring (15.76 ± 2.03 vs 12.15 ± 1.99), interpersonal (15.76 ± 2.08 vs 11.88 ± 1.03), and the total scores (78.80 ± 7.89 vs 60.21 ± 7.44), with statistically significant differences (*P* < 0.001) (Table 2).

**Table 2:** Comparison of scores of self-directed learning ($\overline{{\varvec{\upchi}} }$±S, score)(*N* = 169)

Variables	Observation group (*n* = 84)	Control group (*n* = 85)	t	P	d	95%CI
Learning motivation	23.58 ± 3.47	18.29 ± 2.93	10.71	0.000	1.648	1.297 ~ 1.996
Planing and implementing	23.69 ± 2.69	17.88 ± 2.46	14.65	0.000	2.254	1.866 ~ 2.638
Self-monitoring	15.76 ± 2.03	12.15 ± 1.99	11.67	0.000	1.796	1.437 ~ 2.152
Interpersonal	15.76 ± 2.08	11.88 ± 1.03	12.26	0.000	1.887	1.522 ~ 2.248
Total scores	78.80 ± 7.89	60.21 ± 7.44	15.76	0.000	2.424	2.024 ~ 2.820

### Professional identity questionnaire for nursing students

The research compared the scores in the professional identity questionnaire between the observation group and the control group. In the observation group, the scores of professional self-concept, the benefit of retention and risk of turnover, social comparison and self-reflection, independence of career choice, and total scores were 22.43 ± 3.77, 14.57 ± 2.37, 11.55 ± 1.61, 8.08 ± 1.25, and 63.39 ± 7.87, respectively. At the same time, for the control group, they were 18.05 ± 3.48, 11.75 ± 2.33, 9.56 ± 1.52, 6.58 ± 1.23, and 52.35 ± 7.68, respectively. The research showed that the score of the observation group was significantly higher than the control group (*P* < 0.001). There was no significant difference in social persuasion (*P* > 0.05) (Table 3).

**Table 3:** Comparison of scores of professional identity questionnaire ($\overline{{\varvec{\upchi}} }$±S, score)(*N* = 169)

Variables	Observation group	Control group	t	P	d	95%CI
	(*n* = 84)	(*n* = 85)				
Professional self-concept	22.43 ± 3.77	18.05 ± 3.48	7.86	0.000	1.209	0.879 ~ 1.535
Benefit of retention and risk of turnover	14.57 ± 2.37	11.75 ± 2.33	7.79	0.000	1.198	0.869 ~ 1.524
Social comparison and self-reflection	11.55 ± 1.61	9.56 ± 1.52	8.23	0.000	1.266	0.934 ~ 1.595
Independence of career choice	8.08 ± 1.25	6.58 ± 1.23	7.89	0.000	0.287	-0.161 ~ 0.590
Social persuasion	6.76 ± 1.24	6.41 ± 1.20	1.87	0.063	1.214	0.884 ~ 1.541
Total scores	63.39 ± 7.87	52.35 ± 7.68	9.23	0.000	1.420	1.080 ~ 1.756

### Academic self-efficacy scales

After comparison, the scores of academic self-efficacy scales in the observation group (22.31 ± 3.30) were higher than those in the control group (21.28 ± 2.31, *P* < 0.05) (Table 4).

**Table 4:** Comparison of scores of academic self-efficacy scales ($\overline{{\varvec{\upchi}} }$±S, score)(*N* = 169)

Variables	Observation group	Control group	t	P	d	95%CI
	(*n* = 84)	(*n* = 85)				
Total scores	22.31 ± 3.30	21.28 ± 2.31	2.34	0.021	0.360	0.056 ~ 0.664

### Theoretical scores and practical scores for nursing students

In a comparison of the theoretical scores and the practical scores between the two groups, the observation group was 81.39 ± 3.32 and 93.32 ± 4.70, respectively, while the control group was 76.28 ± 5.90 and 90.67 ± 5.09. The aforementioned data analysis showed that the scores of the observation group were higher than those of the control group (*P* < 0.01) (Table 5).

**Table 5:** Comparison of theoretical scores and practical scores ($\overline{{\varvec{\upchi}} }$±S, score)(*N* = 169)

Variables	Observation group	Control group	t	P	d	95%CI
	(*n* = 84)	(*n* = 85)				
Theoretical scores	81.39 ± 3.32	76.28 ± 5.90	6.95	0.000	1.066	0.742 ~ 1.387
Practical scores	93.32 ± 4.70	90.67 ± 5.09	3.51	0.001	0.541	0.233 ~ 0.847

### Recognition degree for nursing students

A comparison of the recognition degree in learning for the observation group and the control group revealed that teaching method, teacher-student interaction, team cooperation, critical thinking, and communication skills were all improved (*P* < 0.001) (Table 6).

**Table 6:** Comparison of recognition degree(*N* = 169)

Variables	Observation group	Control group	χ^2^	P
	(*n* = 84)	(*n* = 85)		
Teaching satisfaction				
Teaching method(Yes/No)	66/18	32/53	29.05	0.000
Teacher-student interaction(Yes/No)	72/12	34/51	37.76	0.000
Student competence				
Team cooperation(Yes/No)	67/17	39/46	20.74	0.000
Critical thinking(Yes/No)	60/24	31/54	20.78	0.000
Communication skills(Yes/No)	67/17	38/47	21.36	0.000

## Discussion

This research examined the effects of the teaching method combined with situational awareness and case-based seminars in a comprehensive nursing skills practice course. The research found that the comprehensive nursing skills practice course has promise as an educational intervention that improves self-directed learning, professional identity, academic self-efficacy, theoretical scores, practical scores, teaching satisfaction, and student competence. Zhu’s study found that the comprehensive nursing skills course can improve students’ self-confidence and satisfaction [43]. This is similar to our findings. This research provides references to combine situational awareness and case-based seminars to effectively improve the level and quality of nursing teaching efforts.

Nursing is a subject with high application and practicality. Shirazi et al. deemed that self-directed learning helps students learn more by allowing them to study alone, taking personal responsibility for their learning, and mastering the material [44]. These operations in the control group are not case-based exercises, and most students may not know which operations to use when confronted with a patient during their internships. The three assignments that were provided in the observation group increased students’ self-directed learning. They need to decide what experimental operations to conduct and how to conduct them. It is also a demonstration of problem-solving skills and clinical judgment. This process inspired students’ logical clinical thinking and learning motivation and enabled them to identify, analyze, and resolve problems through a range of activities. It promotes the internalization of knowledge, emotional perception, innovative thinking, and critical thinking, all of which are essential for clinical nursing [45]. Tekkol et al. indicated that self-directed learning enhances individual ability through better learning organization, applying new knowledge to more complex and expansive contexts, addressing problems, and active learning [46]. Shahin et al. deemed that nursing students’ self-directed learning and critical thinking could improve when they were teaching with a problem-solving approach [47]. Moving away from the teacher-centered approach to the student-centered integrated approach is a trend in current medical education [48]. Through the preparation of the clinical nursing case materials, case-based learning emphasizes teacher’ guidance to encourage students to form more effective, comprehensive clinical nursing thinking habits [49]. Lecturers will seldom have to worry about students failing to prepare for the planned lesson. Interacting with students who are truly prepared for class activities makes for a more stimulating discussion at a higher level of learning, as opposed to students remaining passive, resulting in an inattentive listener who turns up to a lecture just to sign up for it [50]. At the same time, we have integrated situational awareness into our experimental group instruction. Students will determine what they are going to do based on experience. In Chen’s research, the situational approach could increase students’ motivation to learn, improve students’ communication ability, and broaden students’ knowledge [51]. Li et al. also found that Situational simulation teaching can help to increase the teaching level and learning efficiency [52]. Markus et al. indicated that making use of situational awareness during undergraduate medical training could develop and improve teaching on efficient processing and information gathering [53]. The above studies are partly in accordance with this research. Yu et al. indicated that rising academic self-efficacy levels may improve nursing students’ independence, leading to increased attempts to overcome obstacles, accomplish goals, and eventually improve the quality of care [54]. The improvement of academic self-efficacy can help students adjust their learning attitudes, thereby continuously improving their learning confidence and gradually forming long-term learning abilities.

It is widely recognized that nursing is one of the most emotionally draining and stressful professions due to the wide gap between theory and clinical practice [55]. Professional identity is formed via self-awareness as a nurse, clinical experience, and knowledge of one’s position [56]. Meanwhile, professional identity is the psychological foundation that enables individuals to carry out their jobs effectively and achieve organizational goals [57]. As a result, for nursing students who have not been exposed to clinical practice, appropriate role-playing is necessary in the class. It consists of clinical occurrences or processes, as well as the portrayal of crucial features or behaviors. Students can approximate their feelings of duty and responsibility as medical personnel through this process. This teaching approach and strategy enable students to gain repeated practice and proper instructional instruction in a stress-free atmosphere [58]. This process has the potential to reverse stereotypes associated with nurses and the nursing profession among students, such as nurses being seen as “doctors assistants” and so on. A more positive, supportive clinical learning environment is preferred by students [59]. Students learn to cooperate and communicate with others during this study process, which also improves their critical thinking abilities, which is consistent with Lee AK’s findings [60]. Every student takes part in group critiques, which raises student and participation satisfaction in the classroom. It is worth mentioning that this research assessed the students’ general nursing skills, case analysis, clinical judgment, and ethical decision-making skills in the theoretical and practical exams. Obviously, the observation group has improved in these nursing abilities. Nursing and medical education cannot be complete without clinical practice, which prepares nursing and medical students to apply their theories in the real world [61].

Nursing students were satisfied with the application of the teaching method combined with situational awareness and case-based seminars in a comprehensive nursing skills practice course. Situational awareness and case-based seminars are more consistent with the general goals of medical teaching.

### Limitations and strengths of the study

The research has several strengths. First, this research included 169 participants from different grades, which ensured the accuracy of this research. Second, the measures of self-directed learning, academic self-efficacy, and professional identity were widely applied and validated instruments to thoroughly understand the research questions. Finally, most of the indicators in this research were easy and simple to operate, which can be used in a comprehensive nursing skills practice course, and have great theoretical and practical significance in nursing education.

Several limitations in this research should be noted. First, students need to be prepared for task division and reporting before class, which means adding to their academic burden. Second, this research was conducted at a medical college in China. Therefore, it is difficult for the findings to be generalized to students majoring in nursing in other countries. Finally, there were no longitudinal observations of the participants in this cross-sectional study. Accordingly, future research will consequently enhance these aspects.

## Conclusions

To optimize nursing students’ learning and improve the quality of nursing care, the curriculum of nursing comprehensive experiments should take into account a variety of influencing factors. This research examined the use of the teaching method combined with situational awareness and case-based seminars in a comprehensive nursing skills practice course. This method not only enhanced nursing students’ self-directed learning, professional identity, and academic self-efficacy but also taught teaching satisfaction and student competence. It provides new ideas and approaches for future nursing students to learn in theory and practice.

## Data Availability

The data presented in this study are available on request from the corresponding author. The data are not publicly available due to privacy reasons.
